# Cluster of lifestyle risk factors for stomach cancer and screening behaviors among Korean adults

**DOI:** 10.1038/s41598-023-44470-3

**Published:** 2023-10-16

**Authors:** Thao Thi Kim Trinh, Kyeongmin Lee, Jin-Kyoung Oh, Mina Suh, Jae Kwan Jun, Kui Son Choi

**Affiliations:** 1 Graduate School of Cancer Science and Policy, National Cancer Center, 323, Ilsan-ro, Ilsandong-gu, Goyang, Gyeonggi-do 10408 Republic of Korea; 2 National Cancer Control Institute, National Cancer Center, Goyang, Gyeonggi-do Republic of Korea

**Keywords:** Cancer, Cancer prevention, Cancer screening

## Abstract

This study aimed to investigate clustering patterns of lifestyle risk factors for stomach cancer and examine the association of risk factor clusters with stomach cancer screening adherence. Data from the 2019 Korean National Cancer Screening Survey, an annual cross-sectional nationwide survey, were used. The study population included 3539 adults aged 40–74 years with no history of cancer. Six stomach cancer risk factors, including smoking, drinking, physical inactivity, obesity, meat intake, and salted food intake, as well as stomach cancer screening behaviors, were assessed. The most frequent risk factor for stomach cancer was physical inactivity, followed by smoking in males and high salted food intake in females. Compared with participants subjects with no risk factors, those with three or more risk factors were less likely to adhere to screening guidelines (males: adjusted odds ratio [aOR] = 0.35, 95% confidence interval [CI] 0.23–0.53; females: aOR = 0.32, 95% CI 0.21–0.48). Our findings indicate a disparity in stomach cancer screening, such that those with more risk factors are less likely to get screened. Increasing public awareness, providing behavioral counseling, and targeting high-risk populations for screening interventions are critical for promoting cancer screening adherence and reducing the disparity in cancer screening.

## Introduction

Stomach cancer is a common malignant disease^1^. Each year, approximately 1,000,000 people are diagnosed with stomach cancer worldwide, and about 760,000 patients die from this disease^2^. Moreover, in 2019, 29,493 new cases of stomach cancer, accounting for 11.6% of total cancers, were observed in Korea^3^.

Both primary and secondary prevention measures contribute to preventing stomach cancer. Primary prevention strategies, such as eradicating Helicobacter pylori^4,5^ and modifying unhealthy lifestyle risk factors^6,7^, are the main methods of prevention. Several lifestyle risk factors, including smoking tobacco, drinking alcohol, obesity, unhealthy diets, and physical inactivity have been found to be associated with the initiation and progression of stomach cancer^8–12^. Modifying these lifestyle risk factors can significantly reduce the risk of stomach cancer^6^. For example, smoking cessation has been reported to prevent 11% of stomach cancer cases worldwide^13^ and 19.4% of cases in Korea^14^. However, lifestyle risk factors are not randomly distributed but tend to cluster and interact to exponentially elevate the risk of cancer^15–17^. Therefore, the synergy between lifestyle risk factors plays a role in controlling stomach cancer.

In Korea, stomach cancer has been considered a suitable disease for mass screening since 1999^18^. Specifically, all Koreans aged 40 years and older are recommended to be screened for stomach cancer every other year via upper endoscopy or upper gastrointestinal series (UGIS). According to recent estimates from the Korean National Cancer Screening Program, participating in stomach cancer screening can significantly reduce the stomach cancer death rate by 19–23%^19^. Efforts to motivate stomach cancer screening and modify lifestyle risk factors may hold the most significant promise for reducing stomach cancer incidence and mortality.

Previous studies have highlighted the relationship between several lifestyle risk factors for stomach cancer, such as alcohol consumption^20^ and cigarette smoking^21^, and stomach cancer screening participation. However, to date, no study has assessed the association between multiple lifestyle risk factors and adherence to stomach cancer screening recommendations. Therefore, in the current study, we aimed to identify clustering patterns of six well-established lifestyle risk factors for stomach cancer (ever smoking, heavy drinking, physical inactivity, overweight or obesity, high red or processed meat intake, and high salted food intake) among Korean adults aged 40–74 years and to examine the association of the clusters with adherence to stomach cancer screening.

## Materials and methods

### Data sources and study population

Data for this study were obtained from the 2019 Korean National Cancer Screening Survey (KNCSS), an annual, nationwide, population-based cross-sectional survey conducted since 2004 to determine cancer screening rates among the Korean population^22^. The survey participants were randomly selected through a multi-stage random sampling method that was stratified by geographic area, sex, and age. The sampling frame was based on resident registration population data. Details about the sampling procedure are available elsewhere^22,23^.

A professional research agency conducted face-to-face interviews using a structured questionnaire. They recruited study subjects through door-to-door visits at least three times to ensure that all eligible participants (i.e., males aged 40–74 years and females aged 20–74 years with no history of cancer) had an opportunity to participate. All study participants were provided with a sufficient explanation, and they provided informed consent to participate in the survey.

**Figure 1:**
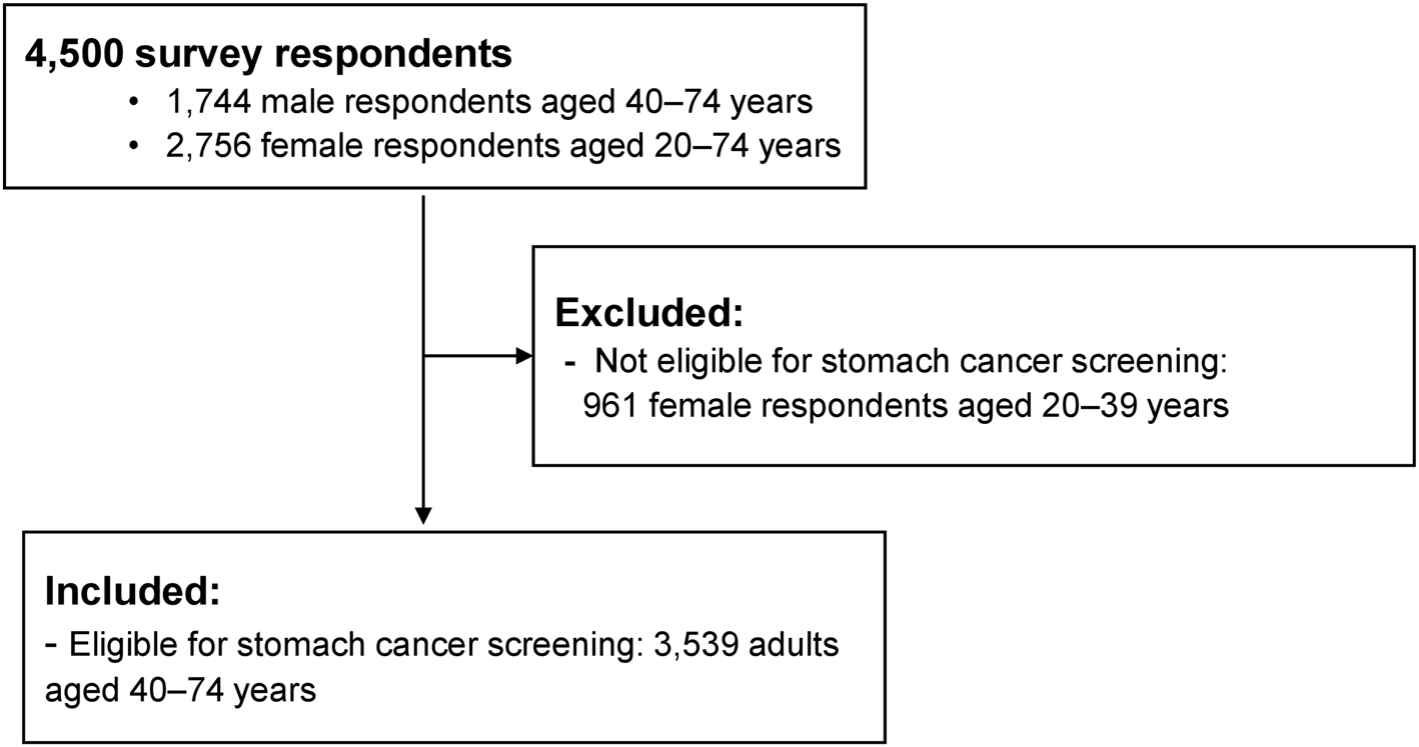
Flowchart of study sample.

In the 2019 KNCSS, participants were asked about their screening history for five types of cancers (stomach, liver, colorectal, breast, and cervical cancer), health behaviors, health status, family history of cancer, and demographic characteristics. Of the 4500 respondents, 3539 adults aged 40–74 years were eligible for stomach cancer screening and included in this study (Fig. 1).

### Measures

#### Lifestyle risk factors

We investigated six lifestyle risk factors based on prior knowledge of risk factors for stomach cancer and public health recommendations^6,8–11^.

##### Ever smoking

Participants were asked about the total number of cigarettes that they had smoked in their entire life (pre-defined categories: “none,” “less than 100 cigarettes,” and “100 cigarettes or more”). We defined ever smokers as those who have smoked at least 100 cigarettes throughout their lifetime^24^.

##### Heavy drinking

Participants answered a series of questions regarding their frequency of alcohol consumption in the past year (“none,” “less than once per month,” “once a month,” “2–4 times per month,” “2–3 times per week,” and “4 or more times per week”) and the number of standard drinks (cups) consumed per each drinking session. A standard drink was defined as a specialized cup for each type of alcoholic beverage (i.e., beer, beer glass; soju, soju shot glass; western liquor, liquor glass; and rice wine, bowl). Each cup has a different volume but a similar amount of alcohol (~ 8 g of pure alcohol)^25,26^. The total daily alcohol consumption amount (g/day) was calculated by multiplying the daily consumption frequency with the amount of alcohol per time. Based on the association between alcohol consumption and the risk of stomach cancer in a previous study among the Korean population^27^, heavy drinking was defined as consuming ≥ 20 g/day for females or ≥ 40 g/day for males.

##### Physical inactivity

Subjects were asked about the number of days they had at least 10 min of moderate-to-vigorous-intensity physical activity during the last week and the number of minutes per day. Moderate-to-vigorous-intensity physical activities include hiking, swimming, shoveling, bicycling fast, basketball game, tennis singles, etc. Information on the total number of minutes per day and the number of days per week was used to calculate the total minutes per week. Participants who did not perform at least 75 min of moderate-to-vigorous-intensity physical activity per week were defined as having “physical inactivity” based on the recommendation of the World Health Organization (WHO)^28^.

##### Overweight or obesity

Based on self-reported height and weight data and using a standard formula for calculating body mass index (BMI, weight [kg]/height squared [m^2^]), individuals with a BMI of ≥ 25 kg/m^2^ were defined as overweight or obese, based on the standard for the general population suggested by the WHO^29^.

##### High red or processed meat intake

We equated 100 g of red and processed meat (including beef, pork, lamb, ham, sausage, bacon, and other processed meats) to one serving and asked participants about the frequency of red and processed meat consumption per week. The frequency was classified into three categories: “one serving per week,” “two to three servings per week,” and “four or more servings per week.” Individuals who reported consuming four or more servings per week were denoted as reporting high red or processed meat intake.

##### High salted food intake

We asked participants about how much they prefer salty flavor using a 5-point Likert scale: (1) “like very much (very salty),” (2) “like (salty),” (3) “neither like nor dislike (neutral),” (4) “dislike (moderate),” and (5) “dislike very much (light).” Individuals who reported “like (salty)” or “like very much (very salty)” were denoted as consuming a high level of preference for salty flavor.

#### Stomach cancer screening status

Participants were asked a series of questions regarding their stomach cancer screening history. The questions were as follows: “Have you ever undergone a UGIS or endoscopy for stomach cancer screening?” “Which screening method did you undergo?” and “When did you last undergo stomach cancer screening with this method?”. Based on the responses, we defined patients as adherent to stomach cancer screening if they had undergone either a UGIS or endoscopy within the last two years, in accordance with the guidelines of the Korean National Cancer Screening Program^30^.

#### Demographic and health-related factors

All participants provided detailed demographic information, including sex, age, education level, monthly household income, and residential area, as well as their health-related status, including their self-perceived health status (good/neutral/bad), comorbidities (having any of the following conditions: hypertension, diabetes, tuberculosis, hepatitis B/C, liver cirrhosis, gastritis, ulcer, colon polyps, benign breast disease, uterine fibroids, hyperlipidemia), and family history of cancer.

### Statistical analysis

The frequency and percentage were used to present the demographic characteristics of the study population, stomach cancer screening status, and six lifestyle risk factors. Chi-squared tests were conducted to determine a difference between two categorical variables. We assigned a binary score (1: yes and 0: no) for each risk factor and estimated the number of risk factors by summing all risk factors reported by each participant (giving a value from 0 to 6). To describe the combinations of each cluster, we used upset diagrams^31^, which visualize complex intersections of a lifestyle risk factor matrix in which the rows represent different sets of combinations, and the columns represent the percentage of participants having these combinations.

Multiple logistic regression analysis was conducted to examine the association between adherence to stomach cancer screening and lifestyle risk factors adjusted for demographic and health-related factors. The outcome was adherence to stomach cancer screening (yes/no), and the independent variables were the six risk factors or multiple lifestyle risk factors, classified into four groups: 0, 1, 2, and 3 + risk factors. The adjusted odds ratio [aOR] and 95% confidence interval [CI] were presented, and the dose–response relationship was examined using a linear trend. Additionally, we performed multinomial logistic regressions to determine the association between adherence to stomach cancer screening according to screening modality and lifestyle risk factors. The odds of having undergone a UGIS or endoscopy was estimated compared with that of not having undergone either procedure. Statistical significance was set at a *p *value < 0.05. All analyses were performed using STATA software version 17 (StataCorp, College Station, TX, USA).

### Ethical considerations

All individuals who enrolled in the 2019 KNCSS provided written informed consent. The study was approved by the Institutional Review Board of the National Cancer Center, Korea (approval number: NCC-2019-0233). This study was conducted in accordance with the principles of the Declaration of Helsinki and the Ethical Guidelines for Medical and Biological Research Involving Human Subjects.

## Results

The sociodemographic characteristics of the study participants from 2019 are presented in Table 1. The distribution of the respondents’ sociodemographic characteristics was similar between males and females, except age and education status. The age distribution of females was higher than that of males, while the education level in males was higher than that in females. The rate of adherence to stomach cancer screening was equal for both sexes (70.8%). Further distribution of the sociodemographic characteristics of the respondents according to stomach cancer screening status is provided in Supplementary Table 1.

**Table 1:** Characteristics of 3539 adults aged 40–74 years in the 2019 KNCSS.

	Total		Males		Females	
	N	%	n	%	n	%
Total	3539	100	1744	49.3	1795	50.7
Age (years)						
40–49	1114	31.5	565	32.4	549	30.6
50–59	1147	32.4	576	33.0	571	31.8
60–74	1278	36.1	603	34.6	675	37.6
Monthly household income (USD)^a^						
< 2000	313	8.8	128	7.3	185	10.3
2000–3999	1301	36.8	702	40.3	599	33.4
≥ 4000	1925	54.4	914	52.4	1011	56.3
Education						
Middle school or below	581	16.4	226	13.0	355	19.8
High school	1849	52.2	823	47.2	1026	57.2
Undergraduate or above	1109	31.3	695	39.9	414	23.1
Residential area						
Metropolitan cities	1546	43.7	751	43.1	795	44.3
Provinces	1993	56.3	993	56.9	1000	55.7
Self-perceived health status						
Good	2393	67.6	1201	68.9	1192	66.4
Neutral	1001	28.3	486	27.9	515	28.7
Bad	145	4.1	57	3.3	88	4.9
Comorbidities						
Yes	1566	44.2	767	44.0	799	44.5
No	1973	55.8	977	56.0	996	55.5
Family history of cancer						
Yes	659	18.6	308	17.7	351	19.6
No	2880	81.4	1436	82.3	1444	80.4
Stomach cancer screening^b^						
Adherent	2504	70.8	1234	70.8	1270	70.8
Non-adherent	1035	29.2	510	29.2	525	29.2

KNCSS, Korean National Cancer Screening Survey; USD, United States dollars.

^a^1USD = 1000 Korean won.

^b^Endoscopy or upper gastrointestinal series within two years preceding.

Table 2 provides the distribution of lifestyle risk factors for stomach cancer. Physical inactivity was the most frequent risk factor in both sexes, followed by smoking in males and high salted food intake in females. More males tended to exhibit clustering of multiple risk factors than females. A cluster of two risk factors was observed in 32.3% of males and 28.1% of females, while a cluster of three or more risk factors was observed in 26.3% of males and 8.7% of females.

**Table 2:** Prevalence of lifestyle risk factors for stomach cancer.

	Total n (%)	Males n (%)	Females n (%)	*p*-value
Risk factors				
Ever smoking				< 0.001
Yes	942 (26.6)	911 (52.2)	31 (1.7)	
No	2597 (73.4)	833 (47.8)	1764 (98.3)	
Heavy drinking				0.303
Yes	91 (2.2)	40 (2.3)	51 (2.8)	
No	3448 (97.4)	1704 (97.7)	1744 (97.2)	
Physical inactivity				< 0.001
Yes	2177 (61.5)	1012 (58.0)	1165 (64.9)	
No	1362 (38.5)	732 (42.0)	630 (35.1)	
Overweight or obesity				0.041
Yes	775 (21.9)	407 (23.3)	368 (20.5)	
No	2764 (78.1)	1337 (76.7)	1427 (79.5)	
High red/processed meat intake				0.013
Yes	426 (12.0)	234 (13.4)	192 (10.7)	
No	3113 (88.0)	1510 (86.6)	1603 (89.3)	
High salted food intake				0.002
Yes	1091 (30.8)	580 (33.3)	511 (28.5)	
No	2448 (69.2)	1164 (66.7)	1284 (71.5)	
Number of risk factors				< 0.001
0	534 (15.1)	214 (12.3)	320 (17.8)	
1	1323 (37.4)	508 (29.1)	815 (45.4)	
2	1067 (30.1)	563 (32.3)	504 (28.1)	
3	442 (12.5)	310 (17.8)	132 (7.3)	
4	148 (4.2)	127 (7.3)	21 (1.2)	
5	23 (0.6)	20 (1.1)	3 (0.2)	
6	2 (0.1)	2 (0.1)	0 (0)	
Median (IQR)	1 (1–2)	2 (1–3)	1 (1–2)	

IQR, Interquartile range.

In the multiple Poisson regression analyses, the number of lifestyle stomach cancer risk factors was significantly decreased in males with education levels above undergraduate and significantly increased in males with comorbidities (Supplementary Table 2).

**Figure 2:**
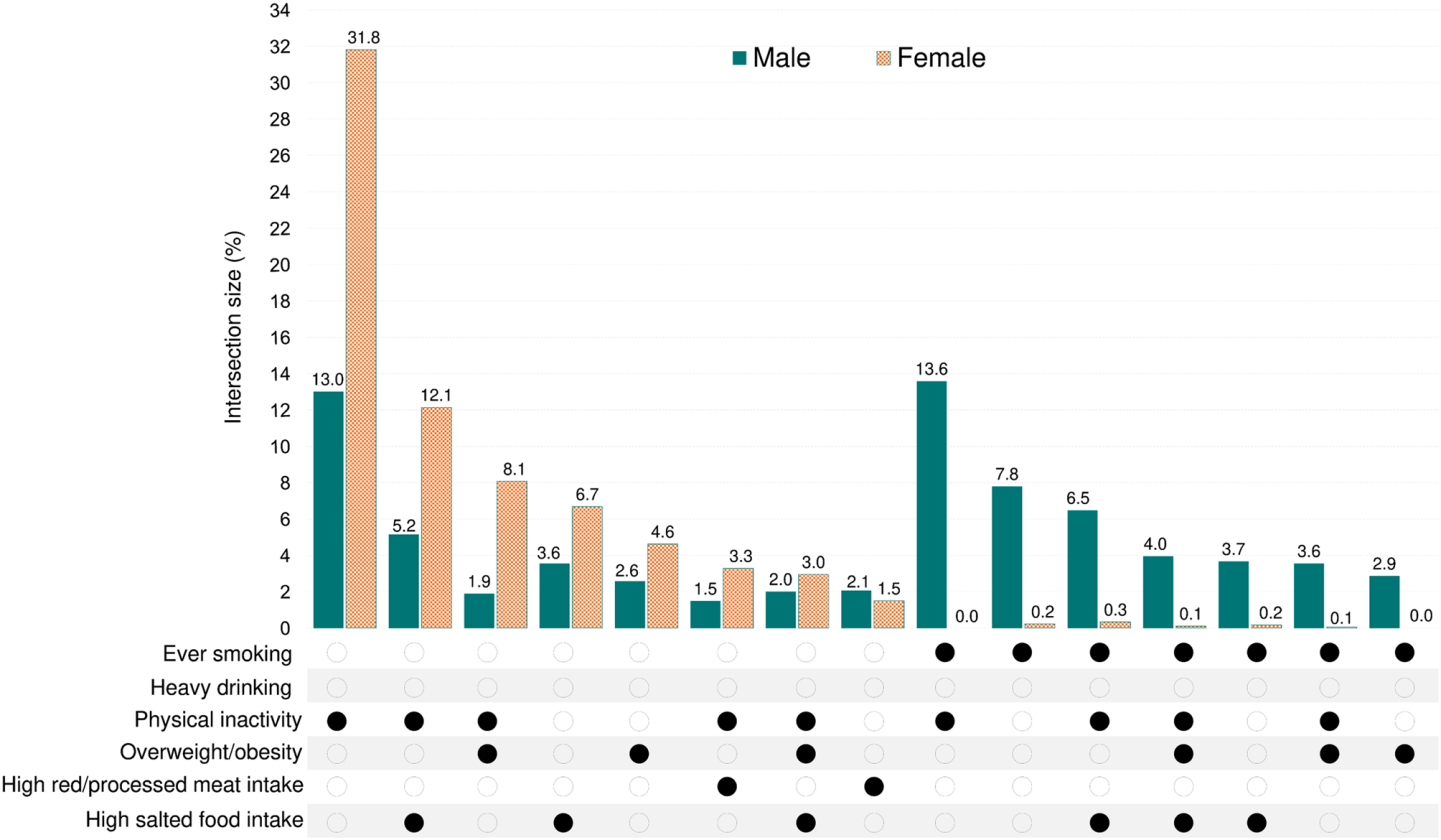
Upset diagram of combinations of lifestyle risk factors for stomach cancer. Note: The combinations with a rate < 1% for both males and females are not shown.

The details of the combination profiles of lifestyle risk factors for stomach cancer are shown in Fig. 2. The most frequent combination among females was physical inactivity and high salted food intake (12.1%), followed by physical inactivity and overweight or obesity (8.1%). In contrast, the combination of smoking and physical inactivity was observed most frequently among males (13.6%). Finally, approximately 6.5% of males had three risk factors: smoking, physical inactivity, and high salted food intake.

Table 3 provides the results of the multiple logistic regression analysis of the association between lifestyle risk factors and adherence to stomach cancer screening. Among males, those who were ever-smokers, were overweight or obese, consumed a high level of red/processed meat, or consumed highly salted food were less likely to adhere to stomach cancer screening. Among females, the lower odds of adherence to stomach cancer screening were observed for those with high red/processed meat or high salted food intake. The dose–response trend for the impact of several lifestyle risk factors on adherence to stomach cancer screening was evaluated in both sexes (p linear trend of < 0.001). Males with more than three risk factors were 65% less likely to adhere to stomach cancer screening (95% CI 0.23–0.53) compared with those without any risk factors. Similarly, females with more than three risk factors were 68% less likely to adhere to stomach cancer screening (95% CI 0.21–0.48) compared with those without any risk factors.

**Table 3:** Multiple logistic regression analysis of the association between lifestyle risk factors and adherence to stomach cancer screening.

	Total			Males			Females		
	Adherent n (%)	Non-adherent n (%)	aOR^a^ (95% CI)	Adherent n (%)	Non-adherent n (%)	aOR^b^ (95% CI)	Adherent n (%)	Non-adherent n (%)	aOR^b^ (95% CI)
Risk factor									
Ever smoking									
Yes	642 (68.1)	300 (31.9)	0.71 (0.58–0.87)**	620 (68.1)	290 (31.9)	0.69 (0.55–0.86)**	22 (71.0)	9 (29.0)	1.00 (0.45–2.22)
No	1862 (71.7)	735 (28.3)	1.00 (reference)	614 (73.7)	219 (26.3)	1.00 (reference)	1248 (70.7)	516 (29.3)	1.00 (reference)
Heavy drinking									
Yes	71 (78.0)	20 (22.0)	1.42 (0.85–2.37)	31 (77.5)	9 (22.5)	1.38 (0.64–2.99)	40 (78.4)	11 (21.6)	1.47 (0.74–2.92)
No	2433 (70.6)	1015 (29.4)	1.00 (reference)	1203 (70.6)	501 (29.4)	1.00 (reference)	1230 (70.5)	514 (29.5)	1.00 (reference)
Physical inactivity									
Yes	1513 (69.5)	664 (30.5)	0.98 (0.84–1.15)	712 (70.4)	300 (29.6)	1.14 (0.91–1.42)	801 (68.8)	364 (31.2)	0.86 (0.69–1.07)
No	991 (72.8)	371 (27.2)	1.00 (reference)	522 (71.3)	210 (28.7)	1.00 (reference)	469 (74.4)	161 (25.6)	1.00 (reference)
Overweight or obesity									
Yes	538 (69.4)	237 (30.6)	0.91 (0.76–1.08)	266 (65.4)	141 (34.6)	0.65 (0.51–0.84)**	272 (73.9)	96 (26.1)	1.28 (0.98–1.68)
No	1966 (71.1)	798 (28.9)	1.00 (reference)	968 (72.4)	369 (27.6)	1.00 (reference)	998 (69.9)	429 (30.1)	1.00 (reference)
High red/processed meat intake									
Yes	143 (33.6)	283 (66.4)	0.14 (0.11–0.18)***	89 (38.0)	145 (62.0)	0.17 (0.13–0.23)***	54 (28.1)	138 (71.9)	0.11 (0.08–0.16)***
No	2361 (75.8)	752 (24.2)	1.00 (reference)	1145 (75.8)	365 (24.2)	1.00 (reference)	1216 (75.9)	387 (24.1)	1.00 (reference)
High salted food intake									
Yes	732 (67.1)	359 (32.9)	0.76 (0.65–0.89)**	387 (66.7)	193 (33.3)	0.73 (0.58–0.91)**	345 (67.5)	166 (32.5)	0.78 (0.62–0.98)*
No	1772 (72.4)	676 (27.6)	1.00 (reference)	847 (72.8)	317 (27.2)	1.00 (reference)	925 (72.0)	359 (28.0)	1.00 (reference)
Number of risk factors									
0	420 (78.7)	114 (21.3)	1.00 (reference)	174 (81.3)	40 (18.7)	1.00 (reference)	246 (76.9)	74 (23.1)	1.00 (reference)
1	982 (74.2)	341 (25.8)	0.85 (0.67–1.09)	378 (74.4)	130 (25.6)	0.75 (0.51–1.13)	604 (74.1)	211 (25.9)	0.92 (0.68–1.26)
2	746 (69.9)	321 (30.1)	0.67 (0.52–0.86)**	404 (71.8)	159 (28.2)	0.63 (0.42–0.95)*	342 (67.9)	162 (32.1)	0.69 (0.50–0.96)*
3+	356 (57.9)	259 (42.1)	0.36 (0.27–0.47)***	278 (60.6)	181 (39.4)	0.35 (0.23–0.53)***	78 (50.0)	78 (50.0)	0.32 (0.21–0.48)***
p-trend^c^			< 0.001			< 0.001			< 0.001

aOR, Adjusted odds ratio; CI, Confidence interval.

^a^Adjusted by sex, age, income level, education, residential area, self-perceived health status, comorbidity, and family history of cancer.

^b^Adjusted by age, income level, education, residential area, self-perceived health status, comorbidity, and family history of cancer.

^c^Linear regression model showing the trend of the number of lifestyle risk factors and adherence to stomach cancer screening.

*p < 0.05, **p < 0.01, ***p < 0.001.

Regarding lifestyle risk factors associated with the adherence of stomach cancer screening by the screening modality, similar results were observed (Table 4). In particular, males with more than three risk factors had 82% and 63% lower odds of adhering to UGIS and endoscopy screening (vs. non-screening participants), respectively. Interestingly, females who were overweight or obese reported higher odds of adhering to UGIS.

**Table 4:** Multinomial logistic regression analysis of the association between lifestyle risk factors and adherence to stomach cancer screening^a^.

	Males		Females	
	UGIS only^b^ (n = 153)	Endoscopy^b^ (n = 1081)	UGIS only^b^ (n = 152)	Endoscopy^b^ (n = 1118)
Risk factors				
Ever smoking (yes vs. no)	0.44 (0.30–0.65)***	0.73 (0.59–0.92)**	1.58 (0.47–5.25)	0.92 (0.40–2.09)
Heavy drinking (yes vs. no)	0.36 (0.05–2.92)	1.54 (0.71–3.34)	0.32 (0.04–2.50)	1.62 (0.82–3.23)
Physical inactivity (yes vs. no)	1.40 (0.95–2.06)	1.10 (0.88–1.38)	0.95 (0.64–1.41)	0.85 (0.67–1.06)
Overweight or obesity (yes vs. no)	0.30 (0.17–0.52)***	0.71 (0,55–0.91)**	2.79 (1.85–4.21)***	1.12 (0.85–1.48)
High red/processed meat intake (yes vs. no)	0.15 (0.07–0.30)***	0.17 (0.13–0.24)***	0.12 (0.06–0.27)***	0.11 (0.08–0.16)***
High salted food intake (yes vs. no)	0.73 (0.49–1.08)	0.73 (0.58–0.91)**	0.77 (0.51–1.16)	0.78 (0.62–0.99)*
Number of risk factors				
0	1.00 (reference)	1.00 (reference)	1.00 (reference)	1.00 (reference)
1	0.94 (0.49–1.78)	0.72 (0.48–1.10)	1.22 (0.69–2.16)	0.90 (0.65–1.23)
2	0.88 (0.47–1.65)	0.60 (0.40–0.90)*	1.21 (0.67–2.19)	0.64 (0.46–0.90)*
3+	0.18 (0.09–0.39)***	0.37 (0.25–0.56)***	0.64 (0.29–1.40)	0.29 (0.19–0.44)***

Values are presented as adjusted odds ratio (95% confidence interval).

UGIS, upper gastrointestinal series.

^a^Adjusted by age, income level, education, residential area, self-perceived health status, comorbidity, and family history of cancer.

^b^Compared with respondents who reported no screening by UGIS or endoscopy within two years prior.

*p < 0.05, **p < 0.01, ***p < 0.001.

## Discussion

This is the first study to investigate the clustering patterns of six lifestyle risk factors for stomach cancer (ever smoking, heavy drinking, physical inactivity, overweight/obesity, high red/processed meat intake, and high salted food intake) and the relationship between the clusters and adherence to stomach cancer screening in Korea using nationally representative survey data. Among all six lifestyle risk factors, physical inactivity was the most frequent risk factor in both sexes, followed by smoking in males and high salted food intake in females. More males tended to exhibit clustering of multiple lifestyle risk factors than females. Specifically, 58.5% of males and 36.8% of females had a cluster of at least two risk factors. Regardless of sex, more lifestyle risk factors were associated with a lower likelihood of adherence to stomach cancer screening. Our study provides significant empirical evidence to guide prevention strategies and cancer screening programs to reduce the burden of stomach cancer.

Previous studies have demonstrated the prevalence of lifestyle risk factors among Korean adults; however, comparisons should be made with caution due to differences in target populations and variations in the investigation and measurement of risk factors. In the present study, physical inactivity was the most frequent risk factor. This finding is consistent with a study that examined five lifestyle risk factors (i.e., smoking status, heavy drinking, obesity, physical inactivity, and unintentional weight loss) in 9945 Koreans aged 45 years and older^32^. This consistent evidence is particularly alarming as insufficient physical activity is a strong risk factor for other types of cancer and non-communicable diseases^33,34^.

In our study, most female participants had one risk factor, whereas clusters of two to three risk factors were more common among males. This finding is consistent with studies performed in other countries^35,36^. The higher prevalence of clustering of risk factors could partially explain why stomach cancer is more common in males^3,37,38^.

We observed a close relationship between the presence of multiple lifestyle risk factors and stomach cancer screening. Compared with those with no risk factors, those with three or more combined risk factors were less likely to adhere to stomach cancer screening guidelines, with an aOR of 0.35 (95% CI 0.23–0.53) in males and 0.32 (95% CI 0.21–0.48) in females. Because this is the first study to assess the association between combined risk factors and stomach cancer screening, we cannot directly compare our results with others. However, a recent study has highlighted the link between composite behavioral risk factors and a lower rate of non-uptake preventive health services, including blood pressure and cholesterol testing, cytology, and mammography^39^. These findings indicate that screening services are provided inequitably, as people with a higher number of lifestyle risk factors are less likely to get screened. There are several possible explanations for this trend. First, people with lifestyle risk factors often take less care of themselves and participate less in screening programs^40^. Second, inadequate knowledge about cancer risk factors and early detection may provide a barrier to adherence to screening guidelines^41^. Finally, the lack of recommendations from health professionals could result in a decrease in participation in screening. Therefore, efforts to improve the cancer screening rate and prevent delays in diagnostic evaluation should address these specific barriers.

Recent research emphasized that stomach cancer is expected to contribute to a substantial number of cases in many countries and is an important cause of mortality if no further action is taken^42,43^. Therefore, more targeted prevention strategies, such as *H. pylori* eradication, smoking control, and healthy diet, should be formulated for people adapting to different genders, age groups, and regions^42^. Moreover, effective cancer-prevention campaigns, such as warning about risk factors and promoting favorable behaviors in the general population, should be formulated. For instance, in Norway, mass media campaigns aimed at colorectal cancer prevention led to an increase in the number of individuals correctly identifying risk factors and expressing willingness to participate in colorectal cancer screening^44^. Further research and surveillance system should be developed to monitor and modify the prevalence of risk factors.

For a correct interpretation of our results, some methodological comments are needed. First, the study utilized cross-sectional data, which limits the ability to establish causation. The relationship could thus be bidirectional, i.e., the uptake of stomach cancer screening might influence lifestyle changes or vice versa. Second, behavioral risk factors and the screening data were self-reported and may reflect either over- or under-reporting and recall biases. Third, in the current study, we could not collect information on the quantity and frequency of salt intake. We could also not use objective measurements such as 24-h urinary Sodium measurement alongside the self-reported salt flavor preference. Relying on self-report may inevitably introduce misclassification and reporting bias. However, a previous study demonstrated a good correlation between self-reported dietary salt intake and a 24-h urine assay of salt.^45^ Lastly, several lifestyle risk factors could not be assessed due to the availability of the data. For example, low fruit consumption^9^, one of the most critical risk factors for stomach cancer development, could not be evaluated.

In conclusion, our results indicate a disparity in stomach cancer screening, in which those who have a more significant number of risk factors are less likely to get screened. Increasing public awareness of lifestyle risk factors and regular cancer screening, providing counseling at the time of screening, and targeting high-risk populations (those with multiple risk factors) for screening interventions are critical to promote cancer screening adherence and motivate health behavior changes. 

### Supplementary Information

Supplementary Tables.

## Data Availability

The data supporting the findings of this study are not publicly available. Nevertheless, these data are available from the corresponding author (Kui Son Choi: kschoi@ncc.re.kr) upon reasonable request.
