# Supplementary material for: Cluster of lifestyle risk factors for stomach cancer and screening behaviors among Korean adults
Source: Sci Rep. 2023 Oct 16;13:17503. doi: 10.1038/s41598-023-44470-3 (PMC10579218; doi:10.1038/s41598-023-44470-3)
Supplement: Supplementary file 1 — Supplementary Tables. [file 41598_2023_44470_MOESM1_ESM.docx]

**Supplementary Materials**

**Cluster of lifestyle risk factors for stomach cancer and screening behaviors among Korean adults**

**Authors**: Thao Thi Kim Trinh, Kyeongmin Lee, Jin-Kyoung Oh**,** Mina Suh, Jae Kwan Jun, Kui Son Choi.

**Supplementary Table 1: Demographic characteristics according to stomach cancer screening status**

|  |  | **Adherent^1^** | |  | **Non-adherent** | |
| --- | --- | --- | --- | --- | --- | --- |
|  |  | **n** | **%** |  | **n** | **%** |
| **Total** |  | 2,504 | 70.8 |  | 1,035 | 29.2 |
| **Sex** |  |  |  |  |  |  |
| Male |  | 1,234 | 49.3 |  | 510 | 49.3 |
| Female |  | 1,270 | 50.7 |  | 525 | 50.7 |
| **Age (years)** |  |  |  |  |  |  |
| 40–49 |  | 780 | 31.2 |  | 334 | 32.3 |
| 50–59 |  | 850 | 33.9 |  | 297 | 28.7 |
| 60–74 |  | 874 | 34.9 |  | 404 | 39.0 |
| **Monthly household income (USD)^2^** |  |  |  |  |  |  |
| <2,000 |  | 172 | 6.9 |  | 141 | 13.6 |
| 2,000–3,999 |  | 880 | 35.1 |  | 421 | 40.7 |
| ≥4,000 |  | 1,452 | 58.0 |  | 473 | 45.7 |
| **Education** |  |  |  |  |  |  |
| Middle school or below |  | 326 | 13.0 |  | 255 | 24.6 |
| High school |  | 1,335 | 53.3 |  | 514 | 50.0 |
| Undergraduate or above |  | 843 | 33.7 |  | 266 | 25.7 |
| **Residential area** |  |  |  |  |  |  |
| Metropolitan cities |  | 1,129 | 45.1 |  | 417 | 40.3 |
| Provinces |  | 1,375 | 54.9 |  | 618 | 59.7 |
| **Self-perceived health status** |  |  |  |  |  |  |
| Good |  | 1,709 | 68.2 |  | 685 | 66.2 |
| Neutral |  | 710 | 28.4 |  | 21 | 28.1 |
| Bad |  | 86 | 3.4 |  | 59 | 5.7 |
| **Comorbidities** |  |  |  |  |  |  |
| Yes |  | 1,152 | 46.0 |  | 414 | 40.0 |
| No |  | 1,352 | 54.0 |  | 621 | 60.0 |
| **Family history of cancer** |  |  |  |  |  |  |
| Yes |  | 496 | 19.8 |  | 163 | 15.7 |
| No |  | 2,008 | 80.2 |  | 872 | 84.3 |

*USD, United States dollars.*

*^1^Endoscopy or upper gastrointestinal series within two years preceding.*

*^2^1USD = 1,000 Korean won.*

**Supplementary Table 2: Associations between the number of lifestyle risk factors for stomach cancer and sociodemographic characteristics of 3,539 adults aged 40-74 years**

| **Variable** | **Total** | |  | **Males** | |  | **Females** | |
| --- | --- | --- | --- | --- | --- | --- | --- | --- |
|  | **Mean (SD)** | **β (95%CI)^1^** |  | **Mean (SD)** | **β (95%CI)^1^** |  | **Mean (SD)** | **β (95%CI)^1^** |
| **Sex** |  |  |  |  |  |  |  |  |
| Male | 1.8 (1.2) | 1.00 (reference) |  | -- | -- |  | -- | -- |
| Female | 1.3 (0.9) | -0.38 (-0.44–-0.33)*** |  | -- | -- |  | -- | -- |
| **Age (years)** |  |  |  |  |  |  |  |  |
| 40–49 | 1.4 (1.1) | 1.00 (reference) |  | 1.7 (1.2) | 1.00 (reference) |  | 1.2 (0.9) | 1.00 (reference) |
| 50–59 | 1.5 (1.1) | -0.01 (-0.08–0.06) |  | 1.9 (1.2) | -0.01 (-0.10–0.09) |  | 1.2 (0.9) | -0.04 (-0.15–0.08) |
| 60–74 | 1.7 (1.0) | 0.01 (-0.07–0.10) |  | 1.9 (1.1) | -0.05 (-0.16–0.06) |  | 1.5 (0.9) | 0.09 (-0.05–0.22) |
| **Monthly household income (USD)** |  |  |  |  |  |  |  |  |
| <2,000 | 1.7 (1.0) | 1.00 (reference) |  | 1.9 (1.1) | 1.00 (reference) |  | 1.6 (1.0) | 1.00 (reference) |
| 2,000–3,999 | 1.6 (1.0) | -0.06 (-0.17–0.04) |  | 1.8 (1.1) | -0.02 (-0.17–0.13) |  | 1.3 (0.8) | -0.10 (-0.24–0.05) |
| ≥4,000 | 1.5 (1.1) | -0.03 (-0.15–0.08) |  | 1.8 (1.2) | 0.05 (-0.11–0.22) |  | 1.2 (0.9) | -0.11 (-0.28–0.06) |
| **Education** |  |  |  |  |  |  |  |  |
| Middle school or below | 1.7 (1.0) | 1.00 (reference) |  | 1.9 (1.0) | 1.00 (reference) |  | 1.5 (0.9) | 1.00 (reference) |
| High school | 1.6 (1.1) | -0.02 (-0.11–0.07) |  | 2.0 (1.2) | 0.05 (-0.07–0.18) |  | 1.3 (0.9) | -0.07 (-0.20–0.06) |
| Undergraduate or above | 1.4 (1.1) | -0.18 (-0.29–-0.07)** |  | 1.6 (1.1) | -0.18 (-0.32–-0.03)* |  | 1.1 (0.8) | -0.15 (-0.33–0.02) |
| **Residential area** |  |  |  |  |  |  |  |  |
| Metropolitan cities | 1.5 (1.1) | 1.00 (reference) |  | 1.8 (1.1) | 1.00 (reference) |  | 1.3 (0.9) | 1.00 (reference) |
| Provinces | 1.6 (1.1) | -0.01 (-0.06–0.05) |  | 1.8 (1.2) | -0.001 (-0.07–0.07) |  | 1.3 (0.9) | -0.02 (-0.10–0.06] |
| **Self-perceived health status** |  |  |  |  |  |  |  |  |
| Good | 1.5 (1.0) | 1.00 (reference) |  | 1.8 (1.1) | 1.00 (reference) |  | 1.2 (0.9) | 1.00 (reference) |
| Neutral | 1.6 (1.1) | 0.01 (-0.05–0.08) |  | 1.8 (1.2) | -0.03 (-0.11–0.05) |  | 1.4 (1.0) | 0.07 (-0.03–0.16) |
| Bad | 1.8 (1.1) | 0.10 (-0.04–0.23) |  | 2.2 (1.2) | 0.11 (-0.08–0.30) |  | 1.6 (1.0) | 0.07 (-0.12–0.26) |
| **Comorbidity** |  |  |  |  |  |  |  |  |
| No | 1.5 (1.0) | 1.00 (reference) |  | 1.7 (1.1) | 1.00 (reference) |  | 1.2 (0.8) | 1.00 (reference) |
| Yes | 1.7 (1.1) | 0.08 (0.02–0.14)** |  | 2.0 (1.2) | 0.09 (0.02–0.17)* |  | 1.4 (1.0) | 0.06 (-0.03–0.16) |
| **Family history of cancer** |  |  |  |  |  |  |  |  |
| No | 1.5 (1.0) | 1.00 (reference) |  | 1.8 (1.1) | 1.00 (reference) |  | 1.3 (0.9) | 1.00 (reference) |
| Yes | 1.6 (1.2) | 0.03 (-0.04–0.09) |  | 2.0 (1.2) | 0.07 (-0.02–0.16) |  | 1.3 (1.0) | -0.04 (-0.15–0.06) |

*SD, Standard Deviation; β, Beta Coefficient; CI, Confidence Interval; USD, United States dollars.*

*^1^Beta coefficient estimated by using Poisson regression, adjusted for all other variables in the table.*

**p < 0.05, **p<0.01, ***p<0.001.*
